# AI AGEISM: A ROADMAP FOR STUDYING AGEISM IN THE ERA OF ALGORITHMS: REFLECTIONS AND FUTURE RESEARCH DIRECTIONS

**DOI:** 10.1093/geroni/igae098.1437

**Published:** 2024-12-31

**Authors:** Justyna Stypinska

**Affiliations:** WZB Berlin Social Science Center, Berlin, Berlin, Germany

## Abstract

AI ageism offers a conceptual framework for analyzing ageism and age discrimination within the field of AI. It encompasses five interconnected forms: (1) age biases in algorithms and datasets (technical level), (2) age stereotypes, prejudices and ideologies of actors in AI (individual level), (3) invisibility of old age in discourses on AI (discourse level), (4) discriminatory effects of use of AI technology on different age groups (group level), (5) exclusion as users of AI technology, services, and products (user level). The presentation reflects on the conceptual framework in the light of empirical evidence and data collected in four countries: Germany, Spain, Great Britain and the Netherlands within an interdisciplinary research project AGEAI. The empirical analysis from three work packages of the project will be shown: a) policy text analysis; b) computational algorithmic simulation; c) qualitative interviews with older persons. The preliminary results substantiate the knowledge about how ageism operates within the field of AI, and expose subtle, yet powerful modes of exclusion of older adults from that field. In conclusion, future directions on research in ageism in AI are discussed

